# Risky anatomical variations of sphenoid sinus and surrounding structures in endoscopic sinus surgery

**DOI:** 10.1186/s13005-022-00336-z

**Published:** 2022-09-03

**Authors:** Gian Luca Fadda, Alessio Petrelli, Anastasia Urbanelli, Paolo Castelnuovo, Maurizio Bignami, Erika Crosetti, Giovanni Succo, Giovanni Cavallo

**Affiliations:** 1 Department of Otorhinolaryngology, University of Turin, San Luigi Gonzaga Hospital, Regione Gonzole 10, Orbassano, 10043 Turin, Italy; 2 National Institute for Health, Migration and Poverty (INMP), Rome, Italy; 3 Department of Otorhinolaryngology, University of Insubria, Varese, Italy; 4 Department of Otorhinolaryngology, Head & Neck Surgery, University of Insubria, Como, Italy; 5 Oncology Department, Head & Neck Surgery, University of Turin, Turin, Italy

**Keywords:** Sphenoid sinus, Internal carotid artery, Optic nerve, Computed tomography, Sellar type, Endoscopic sinus surgery

## Abstract

**Purpose:**

This study aimed to examine the relationship between the sphenoid sinus (SS) and surrounding vital structures such as the internal carotid artery (ICA) and optic nerve canal (ONC) as well as the types of attachment of the sphenoidal septa onto these structures.

**Methods:**

In total, 230 computed tomography (CT) scans were reviewed to study the type of sphenoid sinus pneumatization (SSP), the protrusion and dehiscence of the ICA and ONC, the relationship between the sphenoidal septa and surrounding vital structures as well as pterygoid recess pneumatization (PRP).

**Results:**

The most common SSP was sellar type (58.7%). The rates of protrusion and dehiscence of the ICA were 26.3 and 0.4%, and for the ONC, they were 13 and 1.5%, respectively. The ICA and ONC were most protruded and dehiscent in more extensive SSP. In 21.6% of patients, the intersphenoidal septa (IS) were attached to the wall of the ICA and in 8.6% they were attached to the wall of the ONC. The attachment of IS to the ICA correlated statistically significantly (*p* < 0.0001) with protrusion of the ICA. Accessory septa were detected in 30.4% of cases with various sites of attachment.

**Conclusion:**

To reduce the risk of injury and complications during endoscopic sinus surgery (ESS), surgeons should consider using CT to identify possible bulging and dehiscence of the ICA/ONC and their relationship to the extent of SSP and also to establish the presence of deviation of the sphenoid septum, and the presence of accessory septa.

## Introduction

Understanding the anatomic variations of the sphenoid sinus (SS) using computed tomography (CT) of the paranasal sinuses and their relationship to adjacent neurovascular structures such as the internal carotid artery (ICA) and optic nerve (ONC) is essential to reduce the risk of intraoperative complications during endoscopic sinus surgery (ESS) [1–4].

In the case of extensive sphenoid sinus pneumatization (SSP), these neurovascular structures may be dehiscent or protrude into the air cavities, sometimes without any bone separation. In these cases, they may be susceptible to iatrogenic damage with catastrophic consequences [5]. Moreover, intersphenoidal septa may have attachment points on the bone wall of the ICA and ONC; this represents an anatomical risk factor during ESS, especially in the case of severe fracturing [1, 6].

An accidental fracture of the intersphenoidal septum that attaches itself on the bone wall of the ICA or ONC during endoscopic sinus surgery may result in an injury of these structures, causing severe intraoperative bleeding or blindness.

To the best of our knowledge, this is the first study to perform a multiparametric statistical correlation analyzing 230 computed tomography (CT) scans (460 sides) to investigate the relationship between different patterns of SSP and the surrounding neurovascular structures. Moreover, we evaluated the possible protrusion and dehiscence of these structures, their relationship to a deviated intersphenoidal septum (IS) and accessory septa (AS), and the identification of pterygoid recess pneumatization (PRP).

Verifying the anatomical characteristics of the SS is crucial to reduce the risk of involuntary injury involving these important structures that may occur during ESS.

## Materials and methods

This retrospective study has been performed in accordance with the ethics standards laid down in the 1964 Declaration of Helsinki and informed written consent was obtained from all patients. It included 230 CT scans of paranasal sinuses (460 sides) collected between January 2019 and September 2021. Inclusion criteria were individuals older than 18 years of age with rhinosinusal symptoms. Exclusion criteria were individuals with facial bone fractures, rhinosinusal neoplasms or rhinosinusitis of the posterior paranasal sinus and massive polyposis.

**Fig. 1:**
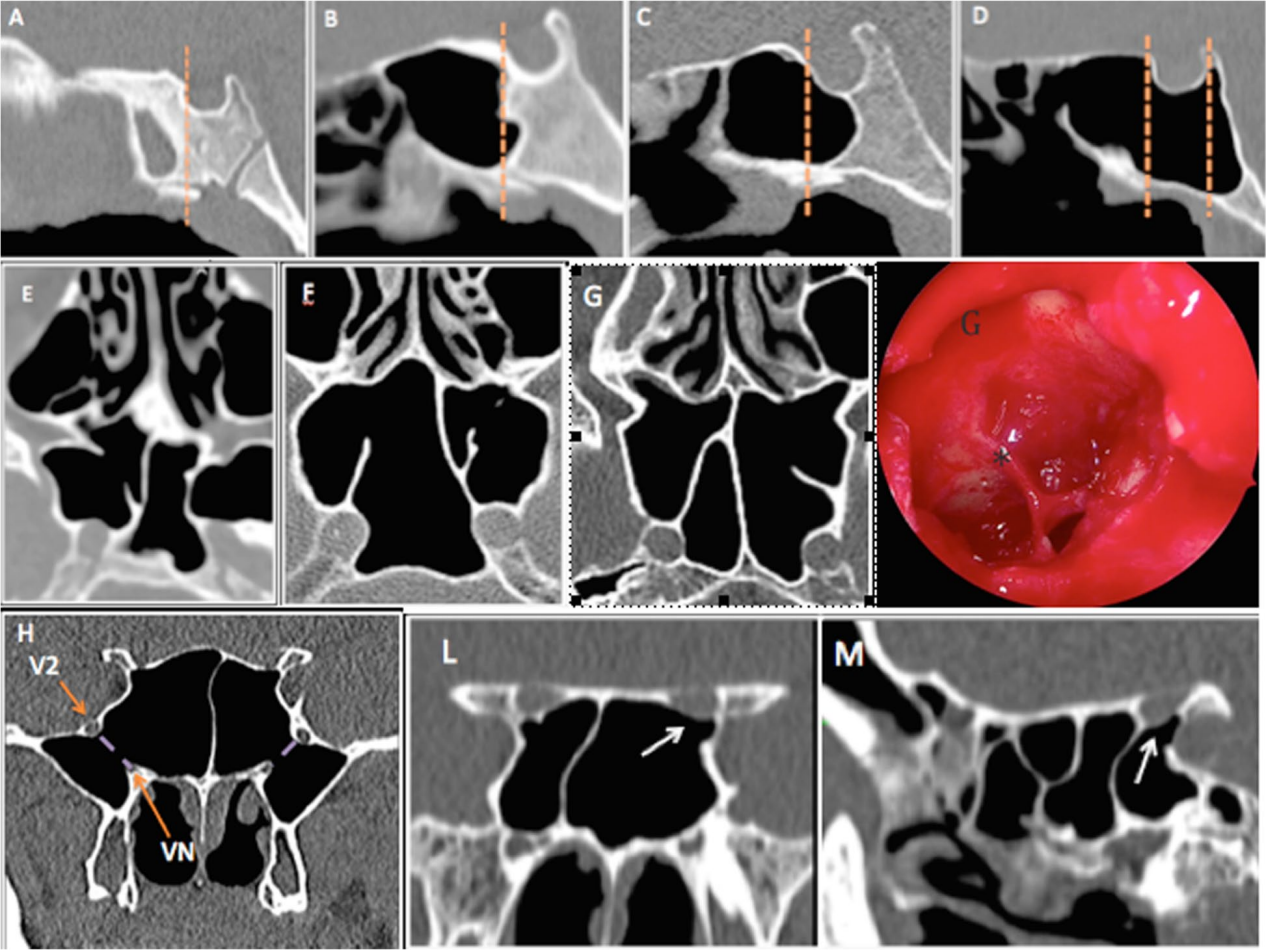
Güldner’s classification^7^ of sphenoid sinus pneumatization on sagittal CT images: **A** conchal, **B** presellar, **C** sellar, and **D** postsellar pneumatization types. Axial CT images showing (**E**) right dehiscent internal carotid artery (ICA) and protrusion of accessory sphenoid septa into the left ICA, **F** deviation of the sphenoid septum in left ICA protrusion, **G** accessory septations with attachment on the right ICA; **G** endoscopic right sphenoid sinus show an accessory septations (*). Coronal (**H**) CT image revealing bilateral pterygoid recess pneumatization. Coronal (**L**) and sagittal (**M**) CT scans showing dehiscence of the left optic nerve (ON) and attachment onto the sphenoid sinus (white arrow)

Based on the CT images, the following variables were assessed: type of SSP, protrusion and dehiscence of the ICA and/or ONC, position and attachment of IS onto these structures, presence of AS and PRP (Fig. 1).

- SSP was evaluated as conchal, presellar, sellar and postsellar types based on the location of the posterior sinus wall with respect to the position of the sella turcica, on the sagittal plane [7]. In conchal-type SSP, pneumatization is absent as there is no association with the sella turcica. In presellar-type SSP, the sinus cavity remains anterior to a vertical line drawn through the tuberculum sellae. In sellar-type SSP, pneumatization extends beyond a vertical line drawn through the tuberculum sellae, whereas in postsellar SSP, pneumatization extends beyond the posterior wall of the sella.
- The ICA and ONC were classified according to their relationship with the sphenoid sinus wall on the coronal plane [8]:
  - protrusion into the air space was defined when the overhang of the neurovascular structure was more than 50% of its circumference [1, 9]
  - dehiscence was defined as the absence of a bony wall separating the ICA/ONC from the sphenoid sinus [10–12]
- IS was analyzed for every scan and classified into three subtypes, on the axial and coronal planes [8]:
  - on the midline; deviation to the right side; deviation to the left side
- Presence of AS, on the axial and coronal planes.
- The incidences of attachment of IS and AS onto the ICA and ONC, protruded and dehiscent, were evaluated individually.
- PRP was defined as a measurable indentation of the bone lateral to a line passing through the center of the vidian canal and the foramen rotundum, on the coronal plane [13].

### Statistical analysis

The associations between variables of interest were evaluated using the Chi-squared test. If the expected frequencies were less than 5 for more than 25% of the cells in the contingency tables, Fisher’s exact test was performed. The analyses were conducted using SAS System 9.4 (SAS Inc., Cary, NC, USA).

## Results

In total, paranasal sinus CT scans from 230 adult patients (460 sides) were analyzed retrospectively. The group comprised 120 men (52.2%) and 110 women (47.8%) whose average age was 53.3 ± 17.1 years with a range of 18–89 years.

No statistically significant differences were identified when the CT scans were grouped according to sex of the patients or the distribution of SSP on the right and left sides.

Analysis of SSP identified a predominance of the sellar type (58.7%), followed by the presellar type (27.2%), conchal type (8.5%) and postsellar type (5.6%) (Table 1). The presence of postsellar pneumatization type on one side was statistically significantly associated with the simultaneous presence of the same pneumatization type on the contralateral side (38.5% for the presence on both sides vs 3.7% for the presence only on the left side; *p* value < 0.0001). Due to the breadth of postsellar sphenoid sinus, the preoperative understanding of this association may represent an advantage in the surgical approach to the pituitary gland.

**Table 1:** Prevalence of sphenoid pneumatization patterns by classification system^7^

Sphenoid Pneumatisation	Right, n 230 (%)			Left, n 230 (%)			TOTAL n 460 (%)	***P***-value*
	Men	Women	Total	Men	Women	Total		
Sellar	62 (48.8)	65 (51.2)	127 (55.2)	77 (53.8)	66 (46.2)	143 (62.2)	**270 (58.7)**	**ns**
Presellar	38 (55.1)	31 (44.9)	69 (30)	27 (48.2)	29 (51.8)	56 (24.3)	**125 (27.2)**	**ns**
Conchal	11 (52.4)	10 (47.6)	21 (9.1)	11 (61.1)	7 (38.9)	18 (7.8)	**39 (8.5)**	**ns**
Postsellar	9 (69.2)	4 (30.8)	13 (5.7)	5 (38.5)	8 (61.5)	13 (5.6)	**26 (5.6)**	**< 0.0001**

*P*-values are referred to the association between right and left side

The ICA was normal in 72.2% and protruded in 26.3% of cases. Isolated ICA dehiscence was detected in only two (0.4%) women and occurred on the left side. In five patients (1.1%), the ICA was dehiscent and protruded simultaneously. The ONC was normal in 83.5%, protruded in 13% and dehiscent in 1.5%. It was protruded in Onodi cells in 2% (Table 2)*.* The presence of normal or protruded ICA (84.4% for the presence on both sides vs 22.8% for the presence only on the left side; *p* value < 0.0001) on one side correlated statistically significantly with a matching presence on the contralateral side (*p* value < 0.0001). The same association was observed for ON (89.6% for the presence on both sides vs 48.6% for the presence only on the left side; *p* value < 0.0001). These correlations could be useful for the surgeon in the approach to the contralateral site during endoscopic sinus surgery.

**Table 2:** Prevalence of neurovascular bulging and dehiscence of surrounding structures

	Right n (%)			Left			Total (***n*** = 460) n (%)	***P***-values*
	Men n (%)	Women n (%)	Total (***n*** = 230) n (%)	Men n (%)	Women n (%)	Total (***n*** = 230) n (%)		
**ICA**								
Normal	92 (53.2)	81 (46.8)	173 (75.2)	83 (52.2)	76 (47.8)	159 (69.1)	332 (72.2)	< 0.0001
Protrusion	28 (51.8)	26 (48.2)	54 (23.5)	36 (53.7)	31 (46.3)	67 (29.1)	121 (26.3)	< 0.0001
Dehiscence	–	–	–	–	2 (100.0)	2 (0.9)	2 (0.4)	< 0.0001
P + D	–	3 (100.0)	3 (1.3)	1 (50.0)	1 (50.0)	2 (0.9)	5 (1.1)	< 0.0001
**ONC**								
Normal	98 (50.8)	95 (49.2)	193 (83.9)	102 (53.4)	89 (46.6)	191 (83)	384 (83.5)	< 0.0001
Protrusion	17 (60.7)	11 (39.3)	28 (12.2)	16 (50.0)	16 (50.0)	32 (13.9)	60 (13)	< 0.0001
Dehiscence	2 (40.0)	3 (60.0)	5 (2.2)	–	2 (100)	2 (0.9)	7 (1.5)	< 0.0001
P in Onodi	3 (75.0)	1 (25.0)	4 (1.7)	2 (40.0)	3 (60.0)	5 (2.2%)	9 (2)	< 0.0001

*ICA* internal carotid artery, *ONC* optic nerve canal, *P* Protrusion, *D* Dehiscence

^*^P-values are referred to the association between right and left side

ICA and ONC were most frequently protruded in sellar and postsellar types on both sides and were dehiscent in the sellar type (Table 3). We found a statistically significant association between left-sided normal (*p* value < 0.001) and protruded (*p* value < 0.01) ICA with types of SS pneumatization, whereas no statistically significant correlation was identified between the ONC and the different types of SS pneumatization. The preoperative knowledge of this correlation should alert the surgeon, since the more pneumatizated the sphenoidal sinus, the greater the risk of injuring vital structures.

**Table 3:** Prevalence of ICA/ONC protrusion and dehiscence and PRP by sphenoid pneumatization

**Sphenoid pneumatization n (%)**	**Right ICA**			**Right ONC**			**Right PRP SS n (%)**^**§**^
	**Normal n (%)**^**§**^	**Protrusion n (%)**^**§**^	**Dehiscence n (%)**^**§**^	**Normal n (%)**^**§**^	**Protrusion n (%)**^**§**^	**Dehiscence n (%)**^**§**^	
Sellar 127 (55.2)	90 (70.9)	34 (26.8)	–	101 (79.5)	21 (16.5)	3 (2.4)	13 (10.2)
Presellar 69 (30)	58 (84.1)	11 (15.9)	–	63 (91.3)	3 (4.3)	2 (2.9)	7 (10.1)
Conchal 21 (9.1)	17 (80.9)	4 (19.0)	–	19 (90.5)	2 (9.5)	–	–
Postsellar 13 (5.7)	8 (61.5)	5 (38.5)	–	10 (76.9)	2 (15.4)	–	–
**Total 230 (100)**	**173 (75.2)**	**54 (23.5)**	**–**	**193 (83.9)**	**28 (12.2)**	**5 (2.2)**	**20 (8.7)**
	**Left ICA**			**Left ONC**			**Left PRP SS n (%)**^**§**^
	**Normal n (%)**^§^**	**Protrusion n (%)**^§^*	**Dehiscence n (%)**^**§**^	**Normal n (%)**^**§**^	**Protrusion n (%)**^**§**^	**Dehiscence n (%)**^**§**^	
Sellar 143 (62.2)	94 (65.7)	49 (34.3)	2 (1.4)	115 (80.4)	22 (15.4)	2 (1.4)	18 (12.6)
Presellar 56 (24.3)	47 (83.9)	9 (16.1)	–	49 (87.5)	6 (10.7)	–	6 (10.7)
Conchal 18 (7.8)	16 (88.9)	2 (11.1)	–	17 (94.4)	1 (5.6)	–	1 (5.6)
Postsellar 13 (5.7)	6 (46.1)	7 (53.8)	–	10 (76.9)	3 (23.1)	–	2 (15.4)
**Total 230 (100%)**	**159 (69.1)**	**67 (29.1)**	**2 (0.9)**	**191 (83.0)**	**32 (13.9)**	**2 (0.9)**	**27 (11.7)**

*ICA* internal carotid artery, *ONC* optic nerve canal, *PRP* pterygoid recess pneumatization, *SS* Sphenoid Sinus

^**^p-value for the association between sphenoid pneumatization and left ICA normal < 0.001

^*^p-value for the association between sphenoid pneumatization and left ICA protrusion < 0.01

^a^% of the total sphenoid pneumatization type (sellar, presellar, conchal or postsellar)

PRP of the SS was identified in 47 (20.4%) patients. It was seen most frequently in the sellar type (Table 3), although no statistically significant correlation was found.

A majority of patients (139/230; 60.4%), both men and women, had a single IS deviated to either the right or left side. Of these patients, a bony IS was attached to the wall of the ICA in 30/139 cases (21.6%) and to the wall of the ONC in 12/139 cases (8.6%). ICA protrusion was associated with right-sided IS in 16.9% and with left-sided IS in 13.2% of cases. Attachments of IS onto the ONC protrusion were noted in 14.3% on both sides and 2.8% on ONC dehiscence (Table 4). We found that attachment of the IS onto the ICA correlated statistically significantly (*p* value < 0.0001) at a higher percentage when it was protruded, as opposed to normal, for both sides. This correlation should alert the surgeon about possible fracture of IS during ESS, resulting in injuries of vital structures like ICA with catastrophic consequences.

**Table 4:** Prevalence of intersphenoid septum and accessory sphenoidal septa

**Intersphenoid septae**	**Men n (%)**^**a**^	**Women n (%)**^**a**^	**Total (*n*** **= 230) n (%)**^**b**^	**Insertion on ICA n (%)**		**Insertion on ONC n (%)**	
Midline	47 (51.7)	44 (48.3)	91 (39.6)	**Protusion**	**Normal**	**Protusion**	**Dehiscence**
Deviate R	35 (49.3)	36 (50.7)	71 (30.8)	12/71 (16.9)	5/71 (7.0)	6/71 (8.4)	2/71 (2.8)
Deviate L	38 (55.9)	30 (44.1)	68 (29.6)	9/68 (13.2)	4/68 (5.9)	4/68 (5.9)	–
**Accessory septae**	30 (13)	40 (17.4)	70 (30.4)	**Insertion on ICA**			
				**R- Protusion** 19/70 (27.1)	**L- Protusion** 26/70 (37.1)	**B- Protusion** 16/70 (22.8)	
				**Insertion on ON**			
				**R- Protusion** 11/70 /15.7)	**L- Protusion** 10/70 (14.3)	**B- Protusion** 7/70 (10)	**R- Dehiscence** 1/70 (1.4)

*R* right, L left, B bilateral, *ONC* optic nerve canal

^a^row percentages

^b^column percentages

AS, in both men and in women, were detected in 70/230 patients (30.4%) with various sites of attachment. On the right side of the SS, the presence of AS was associated with ICA protrusion in 27.1% of individuals and in 15.7% with ONC protrusion. The same association occurred on the left side in 37.1% of individuals with ICA protrusion and in 14.3% with ONC protrusion. AS originating from ON dehiscence was detected in only one case (1.4%). In 10% of cases, the presence of ONC protrusion attaching to AS was bilateral (Table 4). No statistically significant correlation was found for AS.

## Discussion

In advanced ESS, a clear surgical understanding of the anatomic variations in the sphenoid sinus and its pneumatization is helpful since they may place the patient at an increased risk of intraoperative complications with a mortality incidence of about 1% [14].

The Güldner classification defines four types of SSP.^7^ In line with other authors [15–19], the sellar type was the predominant pattern (58.7%) in our study, followed by the presellar type (27.2%). In previous literature, the sellar type is reported in very high percentages ranging between 78.5 and 93% [16–19]. In our study, the conchal type was detected in 8.5% of cases, greater than reported in the literature (1–2%) [17–19]. No conchal type was detected by Wang et al. [20], Dal Secchi et al. [15], or Anusha et al. [21]. The conchal non-pneumatized sphenoid was always considered to be a contraindication for a transsphenoidal approach to the sella [22].

Preoperative CT scans can help to identify bone protrusion of the ICA and ONC into the SS and their dehiscence to help avoid possible injuries during surgery [20]. In our study, ICA protrusion into the SS occurred in 26.3% of cases, similar to Dal Secchi et al. (26%) [15] and Sirikci et al. (26.1%) [10]. Dehiscence of the ICA was identified in only 2 cases (0.4%). In the literature, ICA protrusion generally has a wide range, from 5.2 to 67.0% [9, 16], while its dehiscence ranges from 1.5–5% [1, 15, 16], to 1.5–30% [8, 9, 21].

The rate of ONC protrusion was 13%, in accordance with the literature (range 2.3–35.6%) [8, 9, 16, 21], while dehiscence was 1.5%, lower than the literature (range 0.7–30.6%) [1, 8, 9, 16, 21]. ONC injury by protrusion or dehiscence can occur as a major complication when the IS is attached to it and has to be removed. The risk of injury may lead to defects in the visual field, visual acuity or blindness [11]. The ONC is at greater risk of injury when Onodi cells are present [23]. We found that 2% of our patients presented with the ON protruding into Onodi cells.

Dehiscence and/or protrusion of neurovascular structures are closely associated in cases with high SSP. This should prompt the surgeon to endeavor to preserve them from accidental injury since the bony wall over these structures may be very thin. We found frequent ICA and ONC protrusion in the sellar and postsellar types of SP, similar to earlier studies [15, 24].

Variations in the SS include deviations of the IS and the presence of AS. These are often deviated and attached to the bony wall covering the ICA or ONC. During ESS, care must be taken not to fracture these septa as this may have catastrophic consequences such as uncontrollable bleeding, retrobulbar hematoma, proptosis and diplopia [1, 25]. It has been reported that only one in four IS are located in the midline [26]. In our study, IS was deviated in 60.4% of individuals: in 30.1%, it was attached onto the ICA protuberance and in 14.3% into the ONC protrusion. Poirier et al. [27] reported attachment of the IS onto the ICA in 3.4%, Batra et al. [28] in 37.5% and Dziedzic et al. [26] in 49% of cases. In our work, we identified that 30.4% presented AS similar to levels reported by Anusha et al. [16] Jaworek-Troc et al. [29] reported that AS were present in 78.0% of cases, similar to levels reported by Akgül et al. [17]. Aksoy et al. [25] reported that AS originated in the ICA protuberance in 47.7% of cases while they were associated with the ONC protuberance in 17.5%, comparable to our data. This indicates that deviated IS and AS cause an increased risk of ICA and/or ONC injury; therefore, their presence should be considered during ESS.

The present results and comparison with the relevant literature confirm that risky variants are more likely to occur in the presence of well pneumatized SS. For successful ESS, surgeons should have excellent knowledge of the anatomical relationships present in the sphenoid sinus, and detailed examination of the preoperative CT scans is very important to avoid an increased risk of intraoperative complications. For this purpose, we recommend the use of neuronavigation system in case of important and risky anatomical variations (widely enunciated in this work) of sphenoid sinus and not to use drill and surgical chisel to avoid injuries of vital structures, in particular when approaching IS attaching to protruded ICA or ONC.

## Data Availability

The data used and analyzed during the current study are available from the corresponding author on reasonable request.
